# Targeting ferroptosis in cancer stem cells: A novel strategy to improve cancer treatment

**DOI:** 10.1016/j.gendis.2025.101678

**Published:** 2025-05-09

**Authors:** Luyao Wang, Ye Zhu, Chengying Huang, Qiuming Pan, Junxi Wang, Hongrui Li, Yudi Huang, Guozhong Yi, Zhiyong Li, Songtao Qi, Guanglong Huang, Shanqiang Qu

**Affiliations:** a Department of Neurosurgery, Nanfang Hospital, Southern Medical University, Guangzhou, Guangdong 510515, China; b The First Clinical School of Medicine, Southern Medical University, Guangzhou, Guangdong 510515, China; c Department of Obstetrics and Gynecology, Baiyun Branch, Nanfang Hospital, Southern Medical University, Guangzhou, Guangdong 510440, China; d Nanfang Glioma Center, Nanfang Hospital, Southern Medical University, Guangzhou, Guangdong 510515, China; e Institute of Brain Disease, Nanfang Hospital, Southern Medical University, Guangzhou, Guangdong 510515, China

**Keywords:** Cancer stemcells (CSCs), Cell signaling, Drug target, Ferroptosis, Iron metabolism

## Abstract

Ferroptosis, a distinct regulated cell death process characterized by iron retention and lipid peroxidation, plays a crucial role in the survival of cancer stem cells (CSCs), key contributors to cancer initiation, progression, and recurrence. CSCs exhibit enhanced iron uptake and altered lipid metabolism, allowing them to evade conventional therapies and persist within the cancer microenvironment. Their resilience is linked to low reactive oxygen species levels, aiding survival under oxidative stress. Key regulatory pathways, including the cystine/glutathione axis, significantly modulate CSCs' sensitivity to ferroptosis by maintaining a balance between antioxidant defenses and pro-oxidative stressors. Targeting ferroptosis in CSCs offers promising therapeutic avenues for enhancing treatment efficacy and overcoming resistance. Strategies such as pharmacological inhibition of the SLC7A11 transporter, which reduces cysteine availability and glutathione levels, can potentiate ferroptosis in CSCs. Additionally, inducing dysregulation of iron metabolism or lipid peroxidation can selectively compromise CSCs' survival. Nanoparticle drug delivery systems that increase intracellular iron and reactive oxygen species levels are proving effective in targeting CSCs with minimal impact on normal cells. Ultimately, a comprehensive understanding of the interplay between ferroptosis and CSCs' biology is essential for developing innovative strategies aimed at eradicating these elusive cells, thereby improving cancer treatment outcomes and reducing recurrence rates.

## Introduction

Cancer continues to be a major global cause of death, with rising incidence and mortality rates imposing substantial economic challenges on individuals and society.^1^, ^2^, ^3^ In 2022, global cancer incidence reached approximately 20 million new cases, predominantly driven by lung (12.4%), breast (11.6%), colorectal (9.6%), prostate (7.3%), and stomach cancers (4.9%). Cancer-related mortality totaled 9.7 million deaths, with lung cancer (18.7%) as the leading cause, followed by colorectal, liver, breast, and stomach cancers, while Asia and Africa faced disproportionate burdens due to healthcare disparities and late-stage diagnoses.^4^ Despite notable advancements in cancer research, the prognosis remains suboptimal for many patients. Cancer stem cells (CSCs) significantly contribute to treatment failure by enhancing tumor resilience.^5^, ^6^, ^7^

CSCs, first identified in leukemia by Professor John Dick and colleagues in 1997,^8^ constitute a distinctive subpopulation of cancer cells characterized by self-renewal, differentiation potential, and unlimited proliferative capacity.^9^, ^10^, ^11^ Like normal stem cells, CSCs play pivotal roles in the cancer microenvironment, facilitating cancer initiation, maintenance, and progression.^12^, ^13^, ^14^, ^15^, ^16^ These unique cells can differentiate into various cancer cell types, enhancing both cancer growth and heterogeneity.^17^^,^^18^ Recent research highlights the diverse phenotypes and functions of CSCs, underscoring their pivotal role in recurrence and resistance to therapy.^18^, ^19^, ^20^, ^21^, ^22^, ^23^ While conventional therapies effectively target proliferative cancer cells, CSCs can evade such treatments by entering a dormant state, significantly decreasing metabolic activity and enabling survival without proliferation.^24^, ^25^, ^26^ Upon exposure to extracellular stimuli, these cells can reactivate, regaining the capacity for cell cycle re-entry and proliferation.^27^ This unique biological adaptability results in drug resistance and complicates treatment regimens.^28^, ^29^, ^30^ The self-renewal and therapy resistance of CSCs highlight the critical need for research and novel therapeutic strategies targeting these cells.^31^, ^32^, ^33^, ^34^

Recent research identifies ferroptosis, an iron-dependent, non-apoptotic cell death marked by lipid peroxidation, as a promising field of study.^35^, ^36^, ^37^, ^38^, ^39^, ^40^, ^41^, ^42^ Unlike traditional cell death pathways (*e.g.*, apoptosis and necrosis), ferroptosis is driven by iron-dependent lipid peroxidation and distinct molecular mechanisms.^43^ Recent studies reveal that CSCs exploit ferroptosis resistance mechanisms, such as glutathione (GSH) peroxidase 4 (GPX4) and dihydroorotate dehydrogenase (DHODH) activation, to enhance their survival and drug resistance.^44^ Targeting ferroptosis sensitivity in CSCs could therefore overcome therapeutic challenges in cancer treatment.^45^^,^^46^ Because ferroptosis is closely connected to iron metabolism, research indicates that CSCs exhibit elevated iron concentrations relative to their non-stem counterparts. This altered iron metabolism is essential for sustaining CSCs' characteristics and increases their vulnerability to ferroptosis inducers, offering novel avenues for targeted elimination.^47^, ^48^, ^49^ Further, work by Professor Zhuowei Liu's team demonstrated that CSCs could mitigate sensitivity to ferroptosis, thereby alleviating the effects of chemoradiotherapy.^45^

This review offers an in-depth analysis of ferroptosis characteristics and regulatory mechanisms in CSCs, emphasizing its potential as an innovative cancer treatment strategy. We investigate the impact of ferroptosis on CSCs' survival and drug resistance, focusing on its role in lipid peroxidation and iron metabolism. Future investigations should prioritize optimizing strategies aimed at targeting ferroptosis while also exploring potential synergies with other therapeutic modalities to enhance efficacy and minimize adverse effects. Targeting ferroptosis in CSCs may become crucial in future cancer therapies as scientific advancements progress.

## Ferroptosis and lipid peroxidation

**Figure 1:**
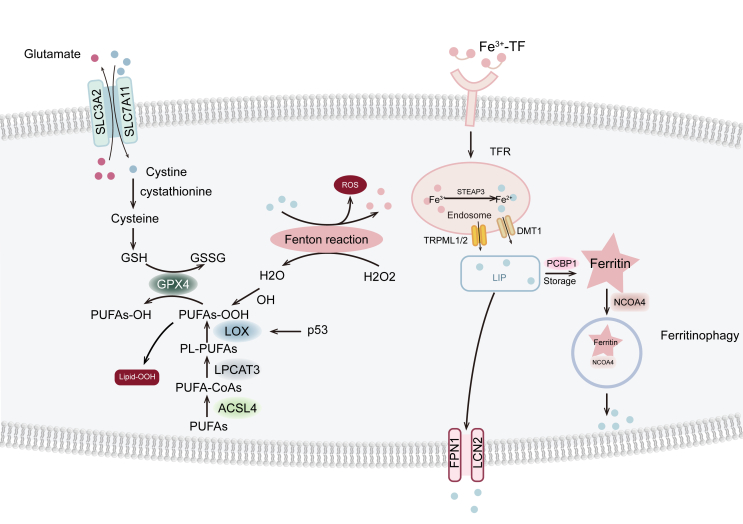
Lipid peroxidation, iron metabolism, and the canonical glutathione peroxidase 4 (GPX4)-dependent pathway are integral components of the ferroptosis mechanism.

Ferroptosis is marked by two hallmark features: iron accumulation and lipid peroxidation.^39^^,^^50^, ^51^, ^52^ Lipids play a crucial role in this type of cell death, where oxidative stress and resulting membrane damage are key factors. Polyunsaturated fatty acids, in particular, exhibit a heightened tendency to undergo peroxidation, promoting ferroptosis.^41^^,^^53^^,^^54^ The regulation of intracellular lipid peroxidation is tightly controlled through two primary pathways (Fig. 1).^54^, ^55^, ^56^ Two primary pathways govern lipid peroxidation within the cell. The first involves the enzymatic conversion of unsaturated fatty acids into highly reactive lipid peroxides.^57^ Arachidonic acid and linoleic acid are primarily metabolized by key enzymes, where acyl-CoA synthetase long-chain family member 4 (ACSL4) activates arachidonic acid to acyl-CoA, which is then esterified with phosphatidylcholine through lysophosphatidylcholine acyltransferase 3 (LPCAT3).^58^^,^^59^ The lipoxygenase (LOX) enzyme family catalyzes lipid peroxidation, and ACSL4 is a key marker for assessing ferroptosis risk. Additionally, ALOX5 and ALOX12 within the LOX family represent key targets for various ferroptosis inducers.^60^, ^61^, ^62^, ^63^

Another critical pathway involved in lipid peroxidation is the Fenton reaction, initiated by free iron ions.^64^, ^65^, ^66^ Iron enters cells mainly via transferrin receptors (TfR), where acidic intracellular conditions release trivalent iron, which is then reduced to bivalent iron by the reductase six-transmembrane epithelial antigen of the prostate 3 (STEAP3).^67^, ^68^, ^69^, ^70^ These iron ions engage in reactions with peroxides, yielding highly reactive radicals responsible for lipid oxidation. Lipid peroxides are kept in balance under normal physiological conditions. A rapid increase in intracellular iron enhances the Fenton reaction, leading to excessive lipid peroxide buildup and triggering ferroptosis.^71^, ^72^, ^73^

## Ferroptosis defense pathways

**Figure 2:**
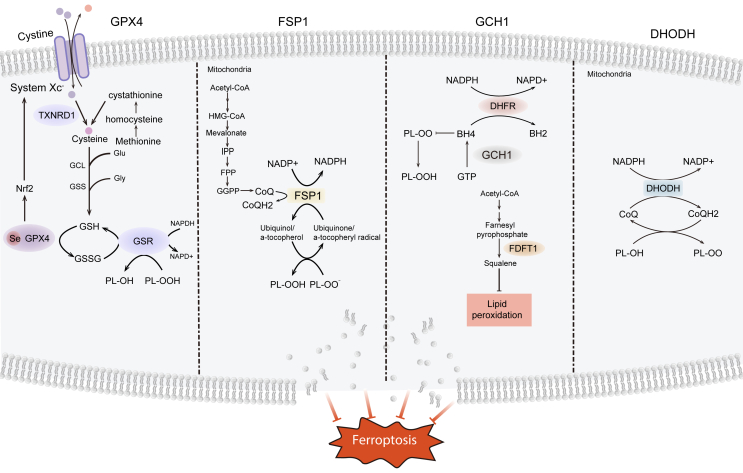
Defense pathways for ferroptosis. Glutathione (GSH) peroxidase 4 (GPX4) catalyzes the reduction of lipid peroxides' oxidative activity in a GSH-dependent manner and is considered a critical factor in mitigating ferroptosis. In contrast, the ferroptosis suppressor protein-1 (FSP1)/coenzyme Q10 (CoQ10) axis serves as a protective mechanism against ferroptosis by independently reducing lipid peroxidation, bypassing the need for GPX4/GSH. The guanosine triphosphate cyclohydrolase 1 (GCH1)/tetrahydrobioterin (BH4) pathway functions as an intrinsic antioxidant system, with GCH1 primarily safeguarding cells from ferroptosis through the antioxidant properties of BH4. Additionally, dihydroorotate dehydrogenase (DHODH) mitigates lipid peroxidation within mitochondria by scavenging free radicals, thereby regulating dihydroubiquinone.

Considerable progress has been made in understanding the mechanisms of enzymatic and non-enzymatic antioxidant defense systems related to ferroptosis.^74^, ^75^, ^76^ Ferroptosis regulation depends on a balance between pathways that produce lipid peroxides and mechanisms that detoxify these byproducts.^77^^,^^78^ Recent research has shown that cancer cells utilize diverse strategies to counteract the harmful effects of lipid modifications (Fig. 2).

### The cystine/GSH/GPX4 pathway

GSH, a tripeptide composed of glutamic acid, glycine, and cysteine, is ubiquitously present in eukaryotic cells.^79^^,^^80^ GSH is present in two forms: reduced (GSH) and oxidized (GSSG). During lipid peroxidation, GSH is converted to GSSG by the action of peroxidases, which neutralize reactive oxygen species (ROS) and inhibit ferroptosis.^81^^,^^82^ GSH is essential for GPX4 function as the main antioxidant in mammalian cells. A lack of GSH directly impairs GPX4 function, heightening vulnerability to ferroptosis.

GPX4, a member of the GPX protein family, is the sole isoform capable of converting phospholipid hydroperoxides into alcohols.^83^, ^84^, ^85^ Genetic removal or pharmacological blocking of GPX4 induces significant lipid peroxidation and initiates ferroptosis in multiple models. GPX4 exists in three distinct isoforms, each with specific subcellular localizations: cytosolic, mitochondrial, and nuclear GPX4, which arise from different transcription initiation sites.^86^^,^^87^ While mitochondrial and nuclear isoforms were initially deemed less critical, recent studies highlight their protective roles against ferroptosis in diverse cellular compartments.^88^^,^^89^ The role of nuclear GPX4 in ferroptosis regulation warrants further investigation.

Cysteine, mainly obtained from cystine through the system *Xc*^*−*^ responsible for cystine transport, acts as the rate-limiting precursor for GSH synthesis.^90^ Cancer cells predominantly obtain intracellular cysteine via solute carrier family 7 member 11 (SLC7A11 or xCT), the transporter component of the system *Xc*^*−*^. Cystine depletion from culture media or pharmacological inhibition of SLC7A11 transport using agents such as erastin or other ferroptosis-inducing compounds effectively induces ferroptosis in diverse cancer cell lines.^91^^,^^92^ The SLC7A11-GSH-GPX4 axis is recognized as a principal defense mechanism against ferroptosis. However, certain cancer cell lines have been shown to retain resistance despite GPX4 inactivation, suggesting that additional defense mechanisms may exist.

### The FSP1/CoQH2 pathway

Initial research focused on GPX4 as the exclusive defense mechanism against ferroptosis.^93^^,^^94^ However, recent studies have identified ferroptosis suppressor protein-1 (FSP1), also known as AIF family member 2 (AIFM2), as a significant player functioning independently of GPX4.^95^ Predominantly localized within lipid droplets and the plasma membrane.^96^^,^^97^ FSP1 reduces ubiquinone (CoQ) to its reduced form, ubiquinol (CoQH_2_), utilizing NAD(P)H, and sequesters lipid peroxidation-derived free radicals, inhibiting lipid peroxidation and ferroptosis.^98^^,^^99^ FSP1 protects against ferroptosis through CoQ, which captures lipid free radicals and regenerates α-tocopherol via its antioxidant properties.^100^ CoQ is mainly produced in mitochondria but is also present in non-mitochondrial membranes like the plasma membrane. The origins of non-mitochondrial CoQ utilized by FSP1 are not yet fully understood. The FSP1-CoQH_2_ pathway independently collaborates with GPX4 and GSH to prevent phospholipid peroxidation and ferroptotic cell death.

### The GCH1/BH_4_ pathway

Guanosine triphosphate cyclohydrolase 1 (GCH1), identified as a ferroptosis suppressor gene via CRISPR genome-wide screening, functions as a rate-limiting enzyme in tetrahydrobiopterin (BH_4_) synthesis, which is a cofactor for aromatic amino acid hydroxylases and other enzymes.^101^, ^102^, ^103^ BH_4_ exhibits robust antioxidant properties, effectively neutralizing free radicals derived from lipid peroxidation and recycling with the assistance of dihydrofolate reductase.^104^ GCH1 regulates ferroptosis by producing BH_4_, a radical-trapping antioxidant, and co-producing CoQH_2_, highlighting the intricate metabolic factors involved in ferroptosis resistance.^101^^,^^105^ Additionally, BH_4_ could promote ubiquinone synthesis by aiding the conversion of phenylalanine to tyrosine, thereby enhancing its antioxidant properties.^106^ In summary, ferroptosis is inhibited through the antioxidant activity of BH_4_ and the production of CoQH_2_ and polyunsaturated fatty acid-rich phospholipids mediated by GCH1. However, the subcellular localization of this system remains to be elucidated.

### The DHODH/CoQH2 pathway

A recent study identified a mitochondria-localized defense mechanism involving DHODH that compensates for GPX4 loss by detoxifying mitochondrial lipid peroxidation.^44^ DHODH, an enzyme on the inner mitochondrial membrane's outer surface, converts dihydroorotate to orotate, producing CoQH_2_.^107^ Following acute inactivation of GPX4, DHODH activity significantly increases, resulting in enhanced CoQH_2_ production that effectively neutralizes lipid peroxides and protects against ferroptosis in mitochondria.^108^

The DHODH-mediated regulation of CoQH_2_ functions as an effective anti-ferroptotic system, analogous to the FSP1 system. Mitochondrial GPX4 and DHODH can compensate for each other to reduce mitochondrial lipid peroxidation, whereas cytosolic GPX4 and FSP1 cannot, likely due to their lack of mitochondrial localization, which limits their ability to detoxify lipid peroxides in the inner mitochondrial membrane.^44^^,^^109^ This discovery highlights the essential function of compartmentalization in protecting against ferroptosis. Further research is necessary to validate this compartmentalization model in the regulation of ferroptosis. Cytosolic GPX4 has been observed to localize significantly within the mitochondrial intermembrane space.^110^ Future research should clarify the presence of cytosolic GPX4 in mitochondria and its potential role in preventing lipid peroxidation in this area.

## Mechanism of inducing ferroptosis in CSCs

**Figure 3:**
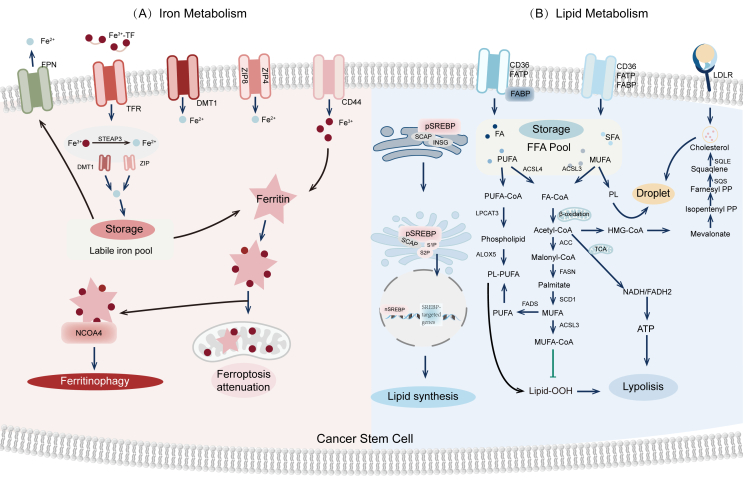
Lipid and iron metabolism in cancer stem cells. **(A)** Divalent metal transporter 1 (DMT1) mediates the entry of reduced iron into the cells, where it is stored in ferritin, used in various metabolic reactions, or delivered to the basolateral membrane for ferroportin-1 (FPN1)-mediated release into the bloodstream. Hp oxidizes Fe^2+^ to Fe^3+^, which can be bound by the iron carrier transferrin (Tf). Cells bind to holo-transferrin through the transferrin receptor (TfR) on their cell surface and internalize this complex via clathrin-dependent endocytosis. Finally, acidic dissociation of Fe^3+^ occurs in endosomes, followed by a six-transmembrane epithelial antigen of the prostate 3 (STEAP3)-mediated reduction to Fe^2+^, which is released into the cytosol via DMT1. **(B)** Cholesterol-rich lipoproteins can be taken up by very-low-density lipoprotein receptor (VLDLR), low-density lipoprotein receptor (LDLR), and lipolysis-stimulated lipoprotein receptor (LSR). The uptake of fatty acids (FAs) through LDLR, fatty acid transport protein (FATP), cluster of differentiation 36 (CD36), and other mechanisms in cancer cells that increase the intracellular FA pool, which results in the plasma membrane exhibiting increased ratio of monounsaturated fatty acid (MUFA) side chains to saturated fatty acid (SFA) side chains and increased ratio of MUFA to polyunsaturated fatty acids (PUFA), thereby preventing ferroptosis. The increase of glycerophospholipids can maintain membrane homeostasis.

Ferroptosis is a regulated cell death process marked by the excessive buildup of lipid peroxides due to disrupted intracellular metabolic pathways.^40^^,^^42^^,^^111^, ^112^, ^113^ This process is associated with both iron metabolism and lipid homeostasis. Prior studies have shown that CSCs have reduced ROS levels, making them more resistant to cell death mechanisms such as ferroptosis.^114^, ^115^, ^116^ Gaining insights into CSC sensitivity to ferroptosis and devising methods to trigger this cell death could significantly advance cancer therapies (Fig. 3).

### Iron homeostasis in CSCs

The cancer microenvironment is defined by numerous stressors, including hypoxia, oxidative stress, and inflammation, all of which present considerable adaptive challenges for CSCs.^117^ Iron acts as a cofactor for iron-dependent enzymes. The involvement of iron in these contexts may constitute a fundamental factor influencing the tumorigenicity of CSCs and their resistance to therapeutic interventions.^118^ Dysregulated iron metabolism is a hallmark characteristic of CSCs, which generally exhibit a higher capacity for iron uptake, reflecting the demands of the cancer microenvironment and their self-renewal capabilities.^48^^,^^49^^,^^119^ This iron uptake is primarily controlled by TfR and ferritin expression levels.^120^, ^121^, ^122^ Disruption in the balance between TfR and ferritin results in deregulated iron metabolism within CSCs.^123^ Various studies have identified significant associations between abnormal iron metabolism and the presence of CSCs across different cancer types.^124^^,^^125^ For instance, glioblastoma and breast cancer CSCs have shown notably increased transferrin expression compared with non-CSCs.^47^ Additionally, research by Schönberg et al highlighted the critical roles of TfR1 and ferritin in maintaining CSCs' functionality *in vivo*.^47^ The importance of iron in regulating CSCs' behavior was first demonstrated in non-small cell lung cancer, where hydroxyl radicals were implicated in aggressive cancer behavior and potential metastasis through up-regulation of SRY-box transcription factor 9 (SOX9).^126^ Tumor-associated macrophages in the tumor microenvironment are crucial “iron donors”, meeting the increased iron requirements of CSCs.^127^ Dextran-modified ferrous-doped hollow mesoporous silica nanoparticles loaded with citrate (DFHC)-regulated tumor-associated macrophages promoted iron release and lipid peroxidation in CSCs via Fe-citrate complexes, inducing ferroptosis through GPX4 suppression and redox imbalance. This mechanism effectively inhibited CSC stemness and tumor initiation, significantly prolonging survival in orthotopic lung cancer models.^128^ Iron supplementation has been demonstrated to enhance CSC-like characteristics in breast, lung, and cholangiocarcinoma cells. Conversely, iron chelation agents inhibited cancer cell globule formation and stemness, as indicated by the expression of surface markers such as cluster of differentiation (CD) 24, CD44, and CD133.^118^^,^^129^ While both chemotherapy and iron deprivation halted cell proliferation in mouse induced pluripotent stem cells, only iron deprivation suppressed stemness marker expression.^130^ This suggests that CSCs require higher iron levels than normal cells during cancer progression, positioning iron homeostasis regulation as a novel strategy for selectively targeting and eliminating CSCs.

### The role of ROS in CSCs

Another significant characteristic of CSCs is their ability to maintain low levels of ROS, which renders them less susceptible to cell death, including ferroptosis. While the understanding of redox homeostasis in CSCs remains nascent, existing studies suggest that, in contrast to conventional cancer cells but akin to normal stem cells, CSCs are characterized by lower levels of ROS.^131^ In various cancer cell types, increased ROS levels, due to oncogene-induced production and reduced antioxidant defenses, trigger pro-tumorigenic signaling pathways.^132^^,^^133^ In contrast, CSCs, exhibiting slower growth rates and diminished oxidative metabolism, produce significantly lower amounts of ROS compared with standard cancer cells. CSCs typically thrive in hypoxic conditions, leading to metabolic adaptations distinct from conventional cancer cells.^46^ Their preference for anaerobic glycolysis significantly reduces ROS production associated with aerobic respiration, minimizing oxidative damage. The preservation of CSC stemness is partly due to low metabolic activity, facilitated by up-regulation of antioxidant genes.^134^ The low ROS levels in CSCs may result from enhanced ROS detoxification, indicated by increased xCT expression and subsequent high GSH production.^135^ Moreover, maintaining low ROS levels mitigates oxidative DNA damage, supports appropriate cell cycle regulation, and decreases CSCs' sensitivity to radiation.^136^ Luo et al were the pioneers in showing that ZMYND8 can reduce ROS levels and lipid peroxidation in breast CSCs.^137^ Ferroptosis inhibition was noted in both mouse models of spontaneous breast cancers and diverse human and mouse breast cancer cell lines. Fluctuations in ROS levels are critical for the reversible transition of CSCs between epithelial and stromal states, significantly influencing cancer metastasis. The stromal state is characterized by low ROS levels, whereas actively metabolizing epithelial CSCs generate higher ROS levels, which promote growth at metastatic sites.^138^ Epithelial CSCs, with their elevated ROS levels, become more reliant on nuclear factor erythroid 2-related factor 2 (NRF2)-mediated redox adaptation. NRF2 not only activates antioxidant responses through the GSH and thioredoxin pathways but also enhances the expression of genes related to drug efflux, stemness maintenance, and cellular plasticity. Epithelial CSCs depend more heavily on NRF2-mediated redox adaptation. Therefore, targeting their adaptation mechanisms could offer a viable therapeutic approach.

### Ferroptosis evasion in CSCs

CSCs are crucial in cancer progression and metastasis.^11^ Although they accumulate elevated labile iron pools due to dysregulated iron metabolism,^47^^,^^139^ they employ various strategies to resist ferroptosis. Lung CSCs enhance SLC7A11 transcription through SRY-box transcription factor 2 (SOX2), leading to ferroptosis resistance.^140^ Breast CSCs utilize a different approach, secreting dickkopf1 to enhance SLC7A11 expression and mitigate ferroptosis, thereby promoting metastatic behavior.^23^ Differentiated CSCs can prevent ferroptosis by elevating deubiquitinase DUBA levels, which stabilize SLC7A11 protein through deubiquitination.^141^ Targeting ferroptosis evasion mechanisms in CSCs could provide valuable insights for cancer therapies focused on eliminating treatment-resistant cells.

The ability to evade ferroptosis plays a crucial role in promoting cancer metastasis. For instance, breast cancer cells exposed to hypercholesterolemic conditions exhibit increased resistance to ferroptosis, resulting in higher metastatic potential.^142^ This resistance may stem from enhanced lipid droplet accumulation and monounsaturated fatty acids within these cells. Melanoma cells initially metastasize regionally through the lymphatic system before spreading systemically via the circulatory system. The lymphatic environment, with elevated oleic acid and GSH levels and reduced labile iron, promotes ferroptosis resistance and supports metastatic survival.^143^

## Advances in targeting ferroptosis in CSCs

**Figure 4:**
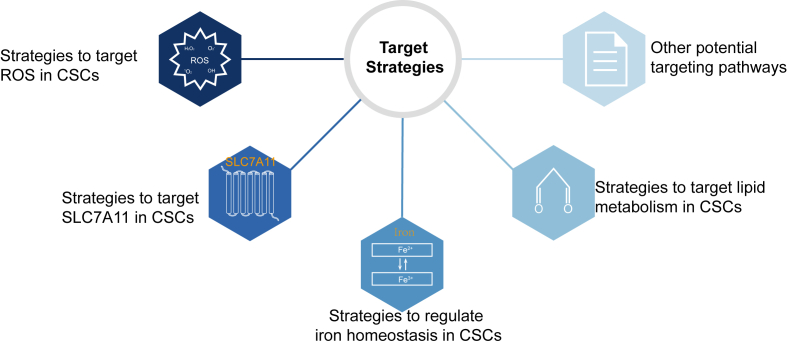
Therapeutic strategies targeting ferroptosis in cancer stem cells (CSCs).

In recent years, multiple strategies aimed at inducing ferroptosis specifically in CSCs have garnered significant interest. Various pharmacological agents have been identified to promote iron-mediated cell death in CSCs, employing distinct mechanisms to regulate intracellular iron levels, enhance ROS production, or modulate associated signaling pathways (Fig. 4).

### Strategies to target ROS in CSCs

An imbalance between ROS generation and removal markedly affects cancer cell viability and responsiveness to drugs.^144^^,^^145^ Redox regulatory therapeutic strategies that target ROS generation and facilitate oxidative stress clearance are gaining momentum. For example, ROS induction by specific compounds leads to reduced stemness in bladder cancer, whereas phenylethyl isothiocyanate (PEITC) depletes GSH, significantly diminishing the side population of colorectal cancer cells *in vitro* and hindering cancer formation *in vivo*.^146^^,^^147^ The combination of PEITC with 5-fluorouracil, as well as doxorubicin with erastin (an xCT inhibitor), synergistically reduces GSH levels and elevates ROS, effectively eliminating breast cancer cells and CSCs.^148^ Increased ROS levels can suppress the hedgehog pathway, making CSCs more susceptible to apoptosis induced by ROS.^149^ Knocking down NRF2 decreases ALDH ^+^ CSCs and mitigates radio-resistance in breast cancer models.^150^ These observations highlight the potential of ROS-targeting strategies in eliminating chemo-resistant and radio-resistant CSCs. Fangchinoline effectively suppresses metastasis in non-small-cell lung cancer by inducing nicotinamide adenine dinucleotide phosphate (NADPH) oxidase 4 (NOX4) degradation, which inhibits cytosolic ROS-mediated protein kinase B (AKT)-mammalian target of the rapamycin (mTOR) signaling and subsequently disrupts epithelial–mesenchymal transition, invasion, and metastasis.^151^

While brusatol, a strong NRF2 inhibitor, effectively suppresses metastasis and reverses drug resistance, it is important to acknowledge the potential negative consequences of inhibiting NRF2.^152^, ^153^, ^154^ As normal cells utilize NRF2 for the detoxification of chemotherapeutic agents, inhibiting it could heighten the adverse effects of these agents. Clinical trials of NRF2 inhibitors have shown limited efficacy, attributed to issues of specificity and potential toxicity.^155^ Remarkably, certain chemotherapeutics, including radiotherapy and cisplatin, can induce oxidative stress, leading to NRF2 activation.^156^, ^157^, ^158^, ^159^, ^160^ Optimizing NRF2 inhibitor and chemoradiotherapy dosing may improve their combined effectiveness, while targeting NRF2 inhibition specifically in cancer cells could reduce toxic side effects in normal tissues.

### Strategies to target SLC7A11 in CSCs

SLC7A11, a critical functional subunit of the cystine/glutamate antiporter system *Xc*^*-*^, serves as a pivotal regulator in ferroptosis modulation through its mediation of cellular cystine transport and redox homeostasis maintenance. Its expression levels are significantly linked to CSCs' susceptibility to ferroptosis, as shown by studies indicating that increased stemness in colorectal cancer correlates with elevated SLC7A11 expression.^161^^,^^162^ SLC7A11 knockdown in these CSCs decreases GSH levels and increases ROS production, thereby inducing ferroptosis.^163^ Investigations into cancer stemness following SLC7A11 knockout revealed marked down-regulation of the stemness marker aldehyde dehydrogenase 1 (ALDH1), alongside reductions in both the size and number of spheroids, highlighting diminished tumorigenicity.^164^ Furthermore, dickkopf WNT signaling pathway inhibitor 1 (DKK1) enhances SLC7A11 expression in breast CSCs, protecting them from lipid peroxidation and ferroptosis.^23^ Therefore, selectively inducing ferroptosis in CSCs through targeting SLC7A11 presents a promising novel approach for cancer therapy.

Vitamin D, recognized for its role in calcium and phosphorus metabolism, has also displayed anti-cancer properties. Research demonstrated that vitamin D reduces SLC7A11 expression in colorectal CSCs, thereby decreasing cysteine uptake and GSH levels, which subsequently induces ferroptosis.^162^ DUB-IN-3 notably promotes liver CSC spheroid formation and inhibits O-GlcNAcylation of the SLC7A11 protein.^165^ The reduced expression of SLC7A11 offers a potential target for cancer treatment, as it could be a useful biomarker for evaluating CSCs' response to ferroptosis-inducing therapies.

### Strategies to regulate iron homeostasis in CSCs

Ferroptosis in CSCs stems from iron homeostasis disruption, where targeted modulation of iron metabolism, specifically augmenting labile iron pools via dual suppression of iron efflux transporters and ROS scavenging systems, enhances therapeutic efficacy.^126^^,^^166^ Nanoparticles offer a method for specifically delivering iron sources or ferroptosis inducers to CSCs, thereby regulating intracellular iron levels and augmenting the effects of ROS inducers.^46^^,^^114^^,^^128^^,^^167^, ^168^, ^169^, ^170^ Luo et al successfully developed a polymer micelle nano-system containing ferrocene, achieving hydrogen peroxide (H_2_O_2_)-responsive cargo release in breast cancer cells, effectively inducing ferroptosis both *in vitro* and *in vivo*.^171^ Liu et al explored multiple iron-based nanomaterials, including Fe_3_O_4_ nanoparticles and metal–organic frameworks.^172^ However, these carriers exhibited significant iron ion leaching. Notably, hydroxy-iron oxide nanoparticles, precursors to Fe_3_O_4_, demonstrated low iron ion leakage rates and high sensitivity to acidic environments. These nanoparticles were utilized to produce FeOOH nanomedicine loaded with siProminin2 and hyaluronic acid. This formulation effectively released Fe^3+^ in the acidic cancer microenvironment, subsequently generating Fe^2+^ through redox reactions with endogenous GSH, leading to increased Fe^2+^ levels and GSH depletion in breast CSCs.^173^ Thus, targeting iron homeostasis regulators in CSCs presents a promising strategy for achieving targeted elimination.

### Strategies to target lipid metabolism in CSCs

Lipid metabolism is crucial in cancer initiation and progression, especially in the proliferation, survival, and migration of CSCs.^174^, ^175^, ^176^, ^177^, ^178^ The metabolic profiles of CSCs differ markedly from those of normal cells, often relying on lipid oxidation and storage via lipid droplets to endure adverse conditions. Previous studies have established that CSCs exhibit an enhanced capability to accumulate lipids, enabling survival in hypoxic and nutrient-deficient environments, thereby utilizing lipid droplets as energy reserves for proliferation.^179^, ^180^, ^181^ CSC functionality is influenced by various lipid metabolic pathways, such as fatty acid synthesis, cholesterol, and phospholipid metabolism.^182^ In CSCs, increased stearoyl-CoA desaturase-1 (SCD1) activity converts saturated fatty acids to monounsaturated fatty acids, facilitating lipid droplet formation and safeguarding CSCs against ROS-induced ferroptosis.^183^ This metabolic adaptation is thought to promote metastasis and recurrence in colorectal cancer. Moreover, Luis et al found that simultaneous inhibition of SCD1 and fatty acid binding protein-4 (FABP4) significantly reduced the proliferation of breast and lung cancer cells.^184^ The uptake and metabolism of polyunsaturated fatty acids are critical for determining cellular sensitivity to ferroptosis. CSCs store lipids in droplets and regulate lipid peroxidation through various metabolic pathways.^176^ Therefore, targeting lipid droplet metabolism may augment lipid peroxide production, promoting ferroptosis. Zhang et al identified that exosomal long non-coding RNA lncFERO from gastric cancer cells enhances SCD1 expression by directly binding to SCD1 mRNA and recruiting heterogeneous nuclear ribonucleoprotein A1 (hnRNPA1).^185^ This interaction leads to polyunsaturated fatty acid dysregulation and inhibition of ferroptosis in gastric CSCs, ultimately contributing to acquired drug resistance. Thus, targeting lipid droplet metabolism in CSCs presents an innovative strategy for inducing ferroptosis, leveraging the unique metabolic characteristics of these cells.

### Other potential targeting pathways

CD44 is essential in controlling ferroptosis, a significant characteristic of CSCs. Isoforms of CD44, particularly CD44v, stabilize the xCT protein, an essential component of the system *Xc*^*−*^, thus facilitating GSH production in gastric cancer.^135^^,^^186^^,^^187^ Additionally, recent studies suggest that the oncogenic protein MUC1-C significantly enhances cancer progression in breast cancer by promoting interactions between CD44v and up-regulated GSH.^188^ Beyond these mechanisms, alternative signaling pathways, including the NRF2 and Hippo-YAP/TAZ pathways, have emerged as potential therapeutic targets influencing ferroptosis susceptibility in CSCs.^189^, ^190^, ^191^, ^192^ The use of ferroptosis inducers has yielded promising results in managing various cancer types, including breast cancer,^193^^,^^194^ ovarian cancer,^195^^,^^196^ and other malignancies.^197^^,^^198^ This emerging evidence underscores the potential of targeting ferroptosis as a novel and effective approach in cancer treatment strategies.

## Challenges and future directions

**Figure 5:**
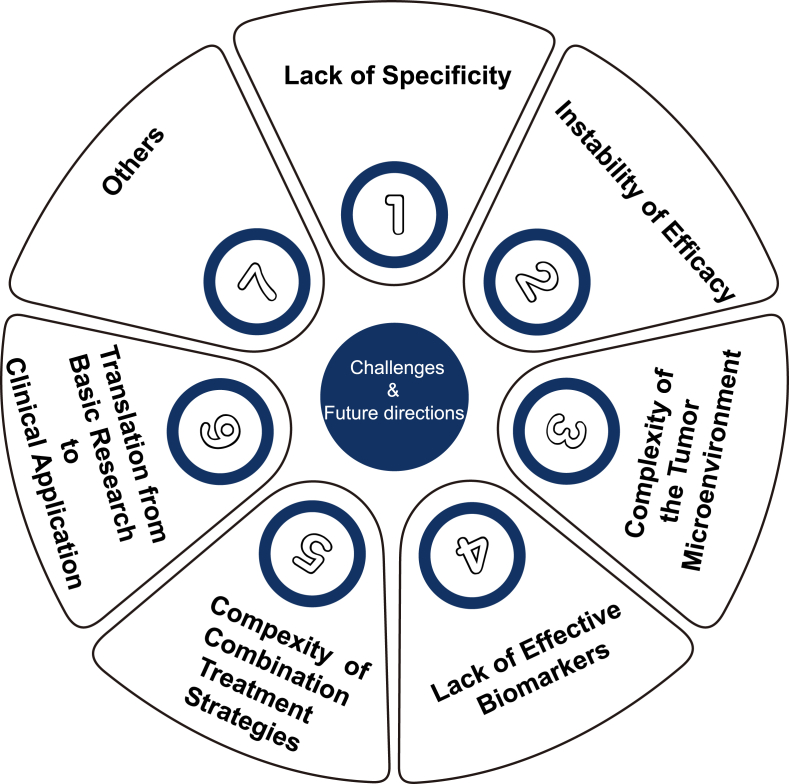
Challenges and future directions in the development of ferroptosis-related therapeutics for cancer treatment.

Inducing ferroptosis in CSCs represents a novel therapeutic strategy in oncology. However, several challenges must be addressed to enhance clinical applications (Fig. 5).

### Lack of specificity

A major obstacle is the lack of specificity among many ferroptosis-inducing agents.^94^ Numerous drugs promoting ferroptosis, such as erastin and RSL3 (a GPX4 inhibitor), primarily target iron metabolism and redox status, with risks extending collateral damage to normal cells at elevated doses, particularly in the liver and kidneys. This lack of selectivity may limit clinical applicability, especially for patients in need of prolonged treatments. Overcoming this challenge will require the identification of new therapeutic targets and the development of selective agents focusing on unique iron metabolic or antioxidant pathways within CSCs. Furthermore, employing nanocarrier systems may enhance the delivery of ferroptosis inducers specifically to CSCs, minimizing toxicity to normal cells through targeted surface modifications.^199^

### Instability of efficacy

The inherent heterogeneity of CSC populations presents another critical challenge.^200^ The variability in sensitivity to ferroptosis-inducing agents among different CSCs can lead to inconsistent treatment responses. Some CSCs may evade ferroptosis by up-regulating antioxidant enzymes, such as GPX, particularly in specific breast cancers and glioblastomas. This underscores the necessity for further research into CSC heterogeneity using advanced single-cell sequencing techniques to discern the characteristics and sensitivities of different subpopulations.^201^ Moreover, combining ferroptosis induction with established therapies such as chemotherapy, radiotherapy, or immunotherapy may mitigate resistance mechanisms and bolster treatment efficacy.

### Complexity of the cancer microenvironment

The cancer microenvironment complicates the induction of ferroptosis. Hypoxic conditions prevalent in cancers activate pathways like hypoxia-inducing factor 1α (HIF-1α), which promote CSCs' survival and proliferation.^202^^,^^203^ Within such environments, CSCs may enhance their antioxidant capacity, further diminishing the efficacy of ferroptosis inducers. Immunosuppressive cells such as tumor-associated macrophages, regulatory T cells, and myeloid-derived suppressor cells can hinder therapeutic effectiveness by suppressing effector T-cell activity and promoting a conducive environment for CSC survival.^204^ Moreover, immune cell secretions may influence CSC iron metabolism, altering sensitivity to ferroptosis.^205^ Targeting hypoxia and immunosuppression through HIF-1α inhibitors and immune checkpoint inhibitors may enhance ferroptosis induction by destabilizing protective features of the cancer microenvironment.

### Lack of effective biomarkers

The absence of reliable biomarkers for predicting CSCs' sensitivity to ferroptosis is a pressing concern. Effective biomarkers are essential for objectively assessing patient responses to treatment, optimizing therapeutic strategies, and minimizing unnecessary side effects. Current CSC markers, including CD44, CD24, and ALDH, exhibit variable expression patterns across different cancer types and lack specificity, complicating the prediction of treatment responses.^206^ Additionally, the dynamic nature of CSCs and their microenvironment can lead to fluctuating expression levels, further complicating predictive efforts. Future research should prioritize the development of functional biomarkers that directly correlate with CSCs' responsiveness to ferroptosis, rather than relying solely on static surface markers. Integrating multiple markers may enhance the accuracy and reliability of sensitivity predictions.

### Complexity of combination treatment strategies

Combination therapies, which incorporate various treatment methods like chemotherapy, radiotherapy, immunotherapy, and targeted therapies, are commonly used to improve therapeutic outcomes.^207^^,^^208^ However, the intricate dynamics inherent in these combinations pose substantial challenges, particularly in terms of targeting CSCs and inducing ferroptosis. CSCs often exhibit robust resistance mechanisms against various therapies, allowing them to evade treatment while conventional cancer cells remain susceptible.^209^ Additionally, patient variability in tolerance to combination therapies can affect treatment continuity and overall effectiveness. Research should prioritize the development of personalized combination strategies while optimizing clinical trial designs to comprehensively evaluate both efficacy and safety.

### Translation from basic research to clinical application

Ultimately, bridging the gap between fundamental research and clinical implementation presents a significant challenge.^210^ Although ferroptosis induction has shown promising results in preclinical studies, these findings often do not translate successfully to clinical settings.^211^ One significant reason for this discrepancy is the reliance on conventional cell lines in basic research, which may not accurately reflect the biological characteristics of patient cancers. Furthermore, animal models frequently fail to replicate the complexities of the human cancer microenvironment, leading to underwhelming results in clinical trials.^212^^,^^213^ Future research should aim to develop advanced cancer models, like 3D culturing systems and organoid models, to more accurately replicate the cancer microenvironment and CSC behavior, thus improving the translational applicability of basic research outcomes.^214^

The exploration of targeting ferroptosis in CSCs holds significant promise, with the potential for numerous breakthroughs and clinical applications on the horizon. Research into ferroptosis mechanisms, novel inducers, and biomarkers will advance therapeutic strategies. Additionally, a focus on combination therapies and the advancement of personalized treatment approaches will be vital. Multicenter, large-scale clinical trials evaluating therapies targeting ferroptosis across diverse cancer types and patient populations, combined with the development of comprehensive clinical databases, are expected to significantly enhance cancer treatment outcomes and offer new hope for patients.

## CRediT authorship contribution statement

Luyao Wang and Chengying Huang: writing-original draft. Ye Zhu: preparation of figures. Qiuming Pan, Junxi Wang and Hongrui Li: collection and analysis of literature. Yudi Huang, Guozhong Yi and Zhiyong Li: review & editing, supervision. Songtao Qi, Guanglong Huang and Shanqiang Qu: conceptualization and the final revision of manuscript.

## Funding

This work was supported by the President Foundation of Nanfang Hospital, Southern Medical University(No. 2022A018), Funding by Science and Technology Projects in Guangzhou (No. 2024A04J5111), and 10.13039/501100021171Guangdong Basic and Applied Basic Research Foundation (No. 2023A1515111129).

## Conflicts of interests

The authors declared no competing interests.
